# Access to Dental Services by People Living with HIV/AIDS: A Cross-Sectional Study in Bahia, Brazil, 2024

**DOI:** 10.3390/dj14070396

**Published:** 2026-07-01

**Authors:** Emanuele Trindade Santos Mota, Sandra Garrido de Barros, Maria Cristina Teixeira Cangussu

**Affiliations:** 1 Professional Master’s Program in Collective Health (MEPISCO), Department of Life Sciences, State University of Bahia, Rua Silveira Martins, 2555, Cabula, Salvador 41150-000, BA, Brazil; 2 Department of Social and Pediatric Dentistry, Faculty of Dentistry, Federal University of Bahia, Rua Araújo Pinho, 72, Canela, Salvador 40110-912, BA, Brazil; cangussu@ufba.br

**Keywords:** health services accessibility, oral health, HIV, AIDS

## Abstract

**Background/Objectives**: This study aimed to assess access to dental services by people living with HIV/AIDS in Bahia, Brazil, and its associated factors. **Methods**: A cross-sectional study was carried out with a convenience sample involving 145 people living with HIV/AIDS in Bahia, recruited from an HIV/AIDS referral center and members of two social organizations. **Results**: Most participants were cisgender men (41.8%), lived in the capital (80.0%), and had a mean age of 44 years (±11.8). More than half had visited a dentist within the previous year (56.2%), mainly for routine or maintenance care (52.4%). Non-disclosure of HIV serological status to dentists was reported by 63.6% of participants and was significantly associated with lower educational attainment (*p* = 0.03). Never having attended a dental appointment was significantly more frequent among black and brown individuals (*p* = 0.04), non-binary participants (*p* = 0.03), and those with fewer years of schooling (*p* = 0.008). **Conclusions**: The results showed inequality in access to oral health care among people with HIV/AIDS in Bahia, influenced by factors such as race, schooling, and gender. Despite economic, geographical, and structural barriers, more than half of the participants had consulted a dentist in the past year and received guidance on the disease’s oral manifestations.

## 1. Introduction

In Brazil, the creation of the National STD/AIDS Program in 1986 marked the consolidation of public policies aimed at combating the epidemic, promoting the establishment of specialized inpatient and outpatient services within the Unified Health System (SUS, in Portuguese, Sistema Único de Saúde) [1]. Guaranteeing free and universal access to antiretroviral therapy (ART) became one of the key milestones in Brazil’s response to HIV/AIDS, earning the country international recognition for the strategies adopted in controlling and treating the disease [2].

Despite this, HIV/AIDS remains a public health challenge in Brazil. Between 2007 and June 2024, 541,759 cases of HIV infection were reported in the country. According to the HIV/AIDS Epidemiological Bulletin for 2024, the annual average of new diagnoses was 36,000 new cases in the last five years [3].

People living with HIV/AIDS have complex and multifaceted health needs that negatively impact their quality of life, well-being, and clinical condition [4]. A lack of financial resources and social support, manifested in difficulties with housing, unemployment, and transportation limitations, poses significant barriers to accessing, maintaining, and continuing care across different levels of the healthcare system [4].

In this context, oral health needs must also be taken into account as they play a key role in the quality of life of people living with HIV/AIDS.

Oral mucosal manifestations associated with HIV may, in many cases, represent the first clinical signs of infection, especially in individuals who have progressed to AIDS. Among the oral lesions most frequently associated with HIV are oral candidiasis, oral hairy leukoplakia, and Kaposi’s sarcoma, lesions that can serve as important indicators of immune compromise and disease progression [5].

Regarding dental assistance for people living with HIV/AIDS, dentists should treat oral lesions, prioritize oral health promotion and prevention efforts, and assess specific vulnerabilities and needs; however, many professionals still do not receive adequate training to care for this population, particularly in areas related to professional ethics, biosafety, and the recognition of oral manifestations associated with immunosuppression [6]. The Brazilian National Oral Health Policy guarantees access to oral health care for the entire population at all levels of the health care system, although barriers to dental care persist due to historically neglected needs.

The increased life expectancy of people living with HIV/AIDS has intensified the demand for oral health care, emphasizing the need for dentists to be trained and committed to providing comprehensive care to this population [7]. However, this growing need comes up against significant barriers to accessing dental services. Studies show that fear of prejudice [8], coupled with experiences of discriminatory practices [9], contributes to perpetuating inequalities and limits the use of these services by this population.

Dentists play a key role in treating oral lesions and promoting oral health among this population. Despite this, this population’s access to dental care is still often hindered by professionals’ lack of knowledge about the disease [10], intersectional stigma, and social determinants [11], factors that impede adequate care, exacerbate oral health inequalities, and negatively affect these individuals’ quality of life.

Adherence to dental treatment is influenced by multiple factors, ranging from objective conditions, such as access to health services and the availability of qualified and trained professionals, to subjective aspects, such as the humanization of care and the strengthening of the bond between patient and professional [6]. In the context of people living with HIV, these elements are often undermined by stigma, prejudice, and negative experiences in health care settings, compromising the continuity and effectiveness of care.

In Brazil, national studies have reached similar conclusions, showing that people living with HIV/AIDS still face discriminatory behavior and various barriers to accessing dental care [7,12]. Research indicates that many individuals avoid disclosing their diagnosis to their dentist out of fear of stigma, discrimination, and social exposure. Added to this are long wait times in the public health system, high costs in the private sector, and limited treatment options, all of which further hinder access to dental care. These findings underscore that prejudice and professional unpreparedness continue to negatively impact the quality of oral health care for this population [12].

In the state of Bahia, no records of studies that have investigated the access of people living with HIV/AIDS to oral health services were found. Therefore, this study describes access to the healthcare network, factors associated with the use of oral healthcare services, and the omission of diagnoses.

## 2. Materials and Methods

This is a cross-sectional, descriptive, and exploratory study conducted with people living with HIV/AIDS in the state of Bahia, Brazil. The strategy for recruiting individuals consisted of applying the data collection instrument in strategic locations that could reach the target audience, including a Specialized Health Service in Bahia and social organizations accompanying people with the disease.

The research participants included adults diagnosed with HIV/AIDS who lived in the state of Bahia, Brazil. Those who did not live in the study area, those under 18, or those who refused to sign the Informed Consent Form were excluded from this study.

A self-administered questionnaire comprising 25 structured questions was developed. The variables included in this study referred to sociodemographic characteristics (date of birth, age, race/color, schooling, work situation, location, sexual orientation and gender), need for dental services (seeking and using them after diagnosis), place sought and/or used (public, private or both), aspects of the consultation (omission of diagnosis) and dental treatment (completion of treatment, time elapsed and reason for last consultation).

The research instrument was developed based on a review of previous studies that used a similar approach, and was pilot-tested for clarity and consistency.

The sample size calculation assumed a finite population and a 50% occurrence of the event—use of dental services in the previous year. There was a need for at least 133 respondents, with a ±5% margin of error, a 95% confidence level, and 80% statistical power.

This study was carried out between April and June 2024 in person at the Specialised State Centre for Diagnosis, Assistance and Research (CEDAP, in Portuguese, Centro Estadual Especializado em Diagnóstico, Assistência e Pesquisa) and by sending messages by email and/or messaging application between members of two social organizations: the National Network of People Living with HIV/AIDS Bahia (RNP+ Bahia, in Portuguese, Rede Nacional de Pessoas vivendo com HIV/AIDS Bahia) and the AIDS Pastoral. During the face-to-face application, users were approached by the researcher herself, who explained this study and invited them to take part.

At CEDAP, the questionnaire was administered 2–3 times a week for three months in the drug dispensing unit, using an iPad or a printed form. The drug dispensing unit was chosen so that the researcher could identify people with HIV/AIDS once they came to pick up their antiretroviral treatment. The researcher did not interfere with participants’ answers, keeping the completion conditions similar to those of participants who completed the questionnaire online. It is important to highlight that in Brazil, access to antiretroviral treatment is guaranteed by the Unified Health System (SUS), and that its sale in private pharmacies is not allowed; so, everybody who uses these drugs receives them through the SUS.

The answers to the printed and digital questionnaires were organized in Microsoft Excel and processed and analyzed using the Statistical Package for the Social Sciences (IBM SPSS Statistics 26.0). Absolute and relative frequencies, measures of central tendency and dispersion, and the chi-square test were used to assess differences between the variables studied. Sociodemographic variables, including sex, skin color, and educational level, were tested for association with the omission of diagnosis by people living with HIV/AIDS using the chi-square test, adopting a 95% significance level (*p* < 0.05).

This study was submitted to and approved by the Research Ethics Committee of the State University of Bahia (UNEB) under protocol number 6728048. All participants signed an informed consent form.

## 3. Results

A total of 145 responses to the questionnaire were recorded, with participants aged 20–68 years, with a mean age of 44 years (±11.8). Self-declared black (47.2%) and brown (37.5%) individuals predominated. As regards gender, 37.3% identified themselves as cisgender women, 41.8% as cisgender men, and 5.2% as people of a non-binary gender. As regards sexual orientation, 47.5% declared themselves to be heterosexual, 38.4% homosexual, and 6.9% bisexual. The majority of participants lived in the capital (80%), while a small proportion (19.4%) lived in municipalities in the interior (Table 1).

As concerns occupations, the most frequent were formal or salaried workers (30%), unemployed (20.9%), and retired or self-employed (16.7% each). Regarding schooling, most respondents had a high school education (42.3%), while the lowest proportion corresponded to those with primary education (6.94%). Participants with higher education and/or postgraduate qualifications accounted for 36.8% of the total (Table 1).

Concerning visits to the dentist, 56.2% of participants reported that their last visit was less than a year ago, with the main reason, regardless of frequency, being routine and/or maintenance visits (52.4%). Regarding guidance on oral health, including prevention and oral manifestations related to HIV/AIDS diagnosis, the percentage of people who had received guidance was almost equivalent to those who had not. However, there was a slight predominance of those who received guidance (50 percent) compared to those who did not (48.6 percent) (Table 2).

Most people (63.6%) said they would disclose their serological status to the dentist, while 33.5% said they had. Among those who did, the main reasons were fear of discrimination (44.6%) and the perception that disclosure was unnecessary for care (36.1%). Table 2 shows the data on the omission of diagnosis and the reasons associated with it.

Most participants used private services (45.9%), while a smaller proportion used public oral health services (34%). Among the 72% who sought dental care after diagnosis, 26.6% reported not receiving treatment due to the unavailability of services in their region and long waiting times, queues, or difficulties in returning.

Most participants (42.7%) completed their last dental treatment, while 38.5% did not. The main reasons for interrupting treatment were lack of financial resources (40.7 percent) and unavailability of service vacancies (40.7 percent).

The association tests showed that the prevalence of people who had never been to a dental appointment was higher among black and brown populations than white populations (*p* = 0.04) (Table 3). The proportion of people who had never been to a dental appointment was also higher among individuals of non-binary gender compared to cisgender women and men. In addition, the group made up of cisgender women and men had a higher frequency of dental consultations carried out less than a year previously compared to the non-binary gender group (*p* = 0.03) (Table 3).

People with complete secondary education, higher education, and/or postgraduate qualifications had their last dental appointment more recently than those with incomplete primary or secondary education (*p* = 0.008) (Table 3). There was an inverse relationship between education level and time elapsed since the last dental appointment: the higher the level of education, the shorter the time elapsed. Similarly, people with a lower level of schooling were more likely to have never seen a dentist or to have done so more recently.

Non-disclosing the diagnosis of HIV/AIDS to the dentist was more frequent among people with complete secondary education, higher education, and/or postgraduate qualifications. On the other hand, the proportion of individuals who did not or would not disclose this information was higher among those with less schooling (*p* = 0.03) (Table 4).

## 4. Discussion

Factors such as race/color, social class, and gender play fundamental roles in structuring society, directly influencing outcomes related to health, illness, and death. In this context, there is a clear correlation between health conditions and social class, in which the processes of illness and poverty cannot be separated [13].

When it comes to the occurrence of HIV/AIDS, socioeconomic and cultural inequalities combined with institutional racism and, in the case of black women, gender-related vulnerabilities [14] are intrinsically related to the increase in the incidence indicators and mortality coefficients of the disease.

The results of this study related to race/color and schooling align with the conclusions of the most recent Brazilian HIV/AIDS Epidemiological Bulletin. The bulletin highlights a significant increase in reported cases among black and brown people, who together account for more than half of all cases (62.8%). In addition, it is also noted that the majority of individuals diagnosed with HIV had completed high school (35.9%) or higher education, either complete or incomplete (22.7%). This scenario is particularly evident in countries with social and health inequalities, like Brazil, where social and racial disparities still constitute essential barriers to comprehensive and equitable health care [15].

In this study, inequality in access to oral health services was also significant regarding gender. People of non-binary genders had less access to oral health services compared to cisgender women and men. The literature has already documented the barriers faced by transgender and non-binary people in accessing and remaining in the health services offered by the SUS; among the main challenges reported is the fragmentation of the healthcare network and structural obstacles [16]. In addition, disrespect for the use of the social name and the prevalence of transphobic attitudes are also pointed out as factors that hinder access to services, aggravating the alienation of these people from the health system [17].

As regards schooling, previous studies on its influence on the occurrence of the disease point to a higher prevalence of HIV/AIDS cases among individuals with fewer years of schooling [18] and show that schooling can act as a protective factor against the disease [19].

Studies analyzing access or barriers to dental treatment for people living with HIV/AIDS have also pointed to the influence of schooling and socioeconomic conditions on the prevalence of the disease [7,12]. The present study found that individuals with complete secondary education, higher education, and/or postgraduate studies had their last dental appointment more recently than those with incomplete primary or secondary education, reaffirming the influence of lower levels of education on dental access.

A study carried out in Fortaleza, Brazilian Northeast, on the use of and satisfaction with public oral health services in the SUS by people living with HIV/AIDS revealed that 76.5% of those interviewed did not disclose their diagnosis at their last consultation [7]. These data align with the present study’s findings, which indicated that most participants would not hide their diagnosis either. In addition, both studies pointed to similar reasons for non-disclosure of the diagnosis among those who chose to hide it, highlighting fear of discrimination or refusal of care as the main reasons.

This research identified an association between non-disclosure of HIV/AIDS diagnosis and the level of education. Non-disclosure was frequent among people who had completed secondary education, higher education, or postgraduate studies. On the other hand, the proportion of individuals who disclosed this information was higher among those with lower levels of education, suggesting that socioeconomic and cultural factors can also influence willingness to reveal the diagnosis to the dentist. Among individuals with higher educational attainment, non-disclosure may also reflect greater concern about confidentiality breaches, fear of subtle forms of discrimination in professional environments and social networks, increased awareness of HIV-related stigma, and a stronger perception that disclosure is unnecessary in routine dental care, particularly when the infection is clinically controlled. Additionally, previous negative experiences within healthcare services and greater autonomy in managing health information may contribute to selective disclosure behaviors.

Difficulty accessing dental treatment was evident in this study, corroborating findings from other studies [7,12]. The main barriers identified were the lack of vacancies and overcrowding in healthcare centers. These results highlight the persistence of structural barriers in the SUS, indicating that many of the challenges faced by the HIV/AIDS population are similar to those faced by the general population in the public health system. This reality contributes to the significant use of private services, as observed in this study and reported in the literature [7,8].

Another study conducted in the city of Chennai, India, which investigated the relationship between the use of dental services, unmet dental needs, and perceived stigma among women living with HIV/AIDS, found that 73.2% of the participants reported difficulty in disclosing their HIV status to their dentist, while 42.9% stated that they had changed dentists after their HIV diagnosis. In addition, more than 73% had unmet dental needs, an association that was statistically significant (*p* = 0.04) [19].

Finally, it is worth noting that there are structural limitations inherent in the health care system itself that significantly influence access to dental services, both for people living with HIV/AIDS and for the general population. Weaknesses related to the availability and organization of care, such as a shortage of professionals, insufficient availability of appointments, longer wait times, and unequal distribution of services, constitute major barriers to timely access and continuity of oral health care, contributing to the persistence of inequalities in the use of dental care [20].

This study’s limitation was the generalization of the findings to rural or more remote areas of Bahia, given the large proportion of participants living in the state capital. Another potential source of bias in this study is its cross-sectional design and reliance on self-reported responses from health service users. Because exposure and outcome were assessed simultaneously, the temporal relationship between variables cannot be established, limiting causal inference. In addition, information bias may have occurred due to participants’ perceptions, recall difficulties, or socially desirable responses. Even so, it was possible to highlight the persistence of inequalities in access to oral health services among people living with HIV/AIDS in Bahia, particularly concerning factors such as race/color, schooling, and gender. Structural barriers in the SUS, such as a lack of places and overcrowding, continue to harm comprehensive care for this population.

## 5. Conclusions

The influence of schooling and socioeconomic conditions on access to dental care is evident: individuals with less schooling and in disadvantaged socioeconomic situations face greater difficulties accessing services. In addition, fear of discrimination and experiences of discriminatory practices emerge as significant obstacles to seeking care.

Although challenges such as the lack of financial resources and the scarcity of places in public health services are persistent, this study also highlighted progress, with more than half of the participants having consulted a dentist in the last year and receiving guidance on the oral manifestations of HIV/AIDS. These results can be related to progress in awareness and access to oral health care for this population, reflecting public policies that promote adequate dental care.

Even so, state health strategies must prioritize reducing these inequalities by expanding the supply of specialized services and raising professionals’ awareness of the importance of oral health care for this population. This effort aims to guarantee accessibility to health services and comprehensive treatment.

## Figures and Tables

**Table 1:** Social and demographic characteristics of HIV/AIDS participants in research, Bahia, Brazil, 2024 (n = 145).

Variables	n	%
**Race/Color**		
White	20	13.8
Black	68	47.2
Brown	54	37.5
Indigenous	2	1.3
Ignored	1	0.1
**Gender**		
Cisgender woman	50	37.3
Cisgender man	56	41.8
Non-binary	7	5.2
Ignored	32	15.7
**Sexual orientation**		
Heterosexual	68	47.5
Homosexual	55	38.4
Bisexual	10	6.9
Ignored	12	7.2
**Locality**		
Capital	116	80.5
Inland Cities	28	19.4
Ignored	1	0.1
**Occupation**		
Salaried worker	43	30.0
Unemployed	30	20.9
Liberal worker	24	16.7
Retired	24	16.7
Domestic worker	6	4.2
Ignored	18	11.5
**Schooling**		
Higher education or postgraduate studies	53	36.8
Secondary education	61	42.3
Elementary Education I *	10	6.9
Elementary Education II *	20	13.3
Ignored	1	0.6

* Elementary Education I: 1st to 5th grades; Elementary Education II: 6th to 9th grades.

**Table 2:** Aspects of dental consultations by people with HIV/AIDS who participated in research, Bahia, Brazil, 2024 (n = 145).

Variables	n	%
**Time since the last appointment**		
Less than a year ago	81	56.2
Between one and two years ago	26	18.0
Three or more years ago	29	20.1
Never been to a dental appointment	3	2.0
Ignored	6	3.6
**Reason for the last appointment**		
Never visited the dentist	2	1.4
Assessment	28	19.5
Initial consultation/first appointment	8	5.5
Routine/maintenance appointment	75	52.4
Urgent/emergency appointment	24	16.7
Ignored	8	4.4
**Oral health guidance after HIV/AIDS diagnosis**		
Yes	72	50.0
No	70	48.6
Ignored	3	1.4
**Has or would non-disclose serological status to the dentist (n = 145)**		
Yes	48	33.5
No	91	63.6
Ignored	6	2.9
**Reason for non-disclosure of HIV/AIDS diagnosis (n = 48)**		
Fear of being discriminated	21	44.6
Fear of not being attended to	2	4.2
Did not consider it necessary	17	36.1
Did not feel confident in the professional	4	8.5
Ignored	4	6.5

**Table 3:** Association between race/color, gender, and schooling with the last dental appointment by people living with HIV/AIDS participants in research, Bahia, Brazil, 2024.

Variable	Categories	Last Appointment at the Dentist			*p*-Value
		Never Used or Used a Long Time Ago n (%)	Less than a Year n (%)	Between 1 and 2 Years n (%)	
Race/Color	White	2 (10.0)	13 (65.0)	5 (25.0)	0.04
	Brown	20 (29.4)	35 (51.4)	13 (19.1)	
	Black	16 (28.0)	33 (57.8)	8 (14.0)	
Gender	Cisgender woman	14 (28.0)	25 (50.0)	11 (22.0)	0.03
	Cisgender man	12 (21.4)	46 (57.1)	13 (21.4)	
	Non-binary	3 (48.8)	4(57.1)	-	
Schooling	Higher education or postgraduate studies	5 (9.4)	36 (67.9)	12 (22.6)	0.008
	Secondary education	13 (29.5)	14 (54.5)	7 (15.9)	
	Elementary Education I *	9 (33.3)	13 (48.1)	5 (18.5)	
	Elementary Education II *	11 (55.0)	7 (35.0)	2 (10.0)	

* Elementary Education I: 1st to 5th grades; Elementary Education II: 6th to 9th grades.

**Table 4:** Association between schooling and non-disclosure of diagnosis by people living with HIV/AIDS participants in research, Bahia, Brazil, 2024.

Schooling	Non-Disclosure of the HIV/AIDS Diagnosis to the Dentist		*p*-Value
	Yes n (%)	No n (%)	
Higher education or postgraduate studies	29 (54.7)	24 (45.3)	0.03
Secondary education	10 (23.3)	33 (76.7)	
Elementary Education I *	7 (25.9)	20 (74.1)	
Elementary Education II *	3 (15.8)	16 (84.2)	

* Elementary Education I: 1st to 5th grades; Elementary Education II: 6th to 9th grades.

## Data Availability

The raw data supporting the conclusions of this article will be made available by the authors on request.

## Abbreviations

The following abbreviations are used in this manuscript:

AIDS	Acquired Immunodeficiency Syndrome
CEDAP	Specialised State Centre for Diagnosis, Assistance and Research, in Portuguese, Centro Estadual Especializado em Diagnóstico, Assitência e Pesquisa
HIV	Human Immunodeficiency Virus
RNP + Bahia	National Network of People Living with HIV/AIDS Bahia, in Portuguese, Rede Nacional de Pessoas vivendo com HIV/AIDS Bahia
SPSS	Statistical Package for Social Science
UNEB	The State University of Bahia, in Portuguese, Universidade do Estado da Bahia
